# Youths’ Habitual Use of Smartphones Alters Sleep Quality and Memory: Insights from a National Sample of Chinese Students

**DOI:** 10.3390/ijerph18052254

**Published:** 2021-02-25

**Authors:** Xiaojing Li, Siqi Fu, Qiang Fu, Bu Zhong

**Affiliations:** 1 Center for Health and Medical Communication, School of Media & Communication, Shanghai Jiao Tong University, Shanghai 200240, China; lixiaojing@sjtu.edu.cn; 2 School of Media & Communication, Shanghai Jiao Tong University, Shanghai 200240, China; fusiqi@sjtu.edu.cn (S.F.); fuqiang-sjtu@sjtu.edu.cn (Q.F.); 3 Donald P. Bellisario College of Communications, Pennsylvania State University, 7 Carnegie, University Park, PA 16802, USA

**Keywords:** habitual smartphone use, sleep quality, memory, social cognitive theory, student

## Abstract

A growing body of work has been devoted to studying the smartphone addiction in youths and its impact on their lives, but less is known about the predictors and effects of youth habitual use of smartphones. Guided by social cognitive theory, this study investigates how habitual smartphone use affects sleep quality and everyday memory based on a nationally representative sample of Chinese students (*N* = 2298). It uses a cluster-randomized sampling with stratification of different areas, consisting of both urban and rural students aged 6–18 years from elementary, middle, and high schools across China. It found that Chinese students exhibited a habitual smartphone use, who were generally confident in using mobile devices, but few had smartphone addiction. Significant gender and age differences were identified concerning the habitual use of smartphone. Specifically, boys demonstrated higher levels of habitual use and smartphone self-efficacy than the girls. High school students showed the highest level of habitual smartphone use compared to those in elementary and middle schools. Smartphone use duration, frequency, and self-efficacy predicted the habitual use, which also led to poorer sleep quality and worse memory outcomes. Prebedtime exposure moderated the relationship between habitual smartphone uses and sleep quality. The results show that students’ habitual smartphone use had a significant impact on their health, cognition and more, even when they exhibited little smartphone addiction. The findings contribute to a better understanding of smartphone impact on school-age youths.

## 1. Introduction

Smartphone use by children and adolescents has been growing at exponential rates around the world, including China [1]. Due to the surge of mobile technology, adolescents spend more and more time on their smartphones every year [2,3]. Research has indicated that 64.2% of elementary school students, 71.3% of middle school students, and 86.9% of high school students have access to a mobile phone in China [4]. Many adolescents report that they “*can’t live without a smartphone*” ([5], p. 725), which is a serious concern for parents and teachers worried about children’s complex relationship with mobile technology.

Prior studies have often treated youth smartphone use like internet addiction behavior, but this may not apply to most young users. A meta-analysis reported that the average prevalence for internet addiction was only 7.02% of the online population, which was based on 113 epidemiological studies with 693,306 subjects from 31 countries that were published in 1996 to 2018 [6]. Similarly, after analyzing 41 studies involving 42,000 children and young people’s smartphone usage, another meta-analysis study discovered that less than a quarter of them (23%) exhibited a prevalence of smartphone addiction [7]. These studies encourage us to explore the habitual use of smartphone among youths, rather than smartphone addiction.

Habits are defined as “*mental and behavioral sequences that occur automatically in presence of a trigger*” ([8], p. 1). A habit can be activated or reinforced as a learned automatic response by a variety of contextual cues, including the environment, other people, or preceding actions in a sequence [9]. Both the usage time and frequency arise as reliable explanations for habitual use of smartphone, which can be affected by the students’ age and gender [10,11,12]. According to social cognitive theory (SCT) [13,14], human behavior is shaped and controlled by both personal cognition (e.g., expectations, beliefs) and environmental cues. Self-efficacy is one of major cognitive force guiding human behavior, which refers to one’s beliefs about the capacity of performing certain tasks in order to accomplish specific goals [14]. In this study, smartphone self-efficacy refers to students’ beliefs in their capability to make good use of the smartphone, who expect positive outcomes in actual smartphone use. Thus, usage time, usage frequency and self-efficacy of smartphone will be explored as potential predictors of youths’ habitual smartphone use.

Considerable research has examined the adverse effects of excessive smartphone use, such as poor sleep quality [15] or worse memory performance [16]. Van den Bulck found that smartphone overuse could disturb students’ sleeping patterns [17]. Even at the time they supposed to be sleeping, those addicted to text messaging would feel uneasy after losing contacts with friends [18]. Compared to those who used smartphones at midnight, those who never did this felt less tired during the daytime [19]. This finding implies that mobile phone use before bedtime can predict increased tiredness due to limited or disturbed sleep at night. Despite the negative impact on sleep quality, smartphones can also be a two-edged sword for youths’ memory functions. For many students, smartphones can be memory extenders and a handy storage device for facts and information [20], but too much reliance on smartphones might have a negative and lasting impact on users’ cognitive abilities, including memory [21]. Research has also identified a significant positive correlation between sleep and memory [22]. Sleep was essential to memory consolidation, during which newly acquired short-term memories are transformed into more stable and long-term memories [23]. Given the high rates of phone use among Chinese students, it’s critical to examine how prebedtime exposure to smartphones may affect their sleep quality and memory.

The impact of habitual smartphone use on youth health is largely understudied in the existing body of scholarship that has several limitations. Firstly, less is known about what causes the habitual use of smartphone and how habitual smartphone use may affect sleep quality and everyday memory. When research solely centers on smartphone addiction, it could neglect the big picture of actual smartphone use in youth. Secondly, few studies have examined the complexity and dynamics of smartphone use with a theoretical model, making the selection of variables used arbitrary. Thirdly, most studies often used convenience samples of college students, rather than a national sample consisting of both children and adolescents.

**Figure 1:**
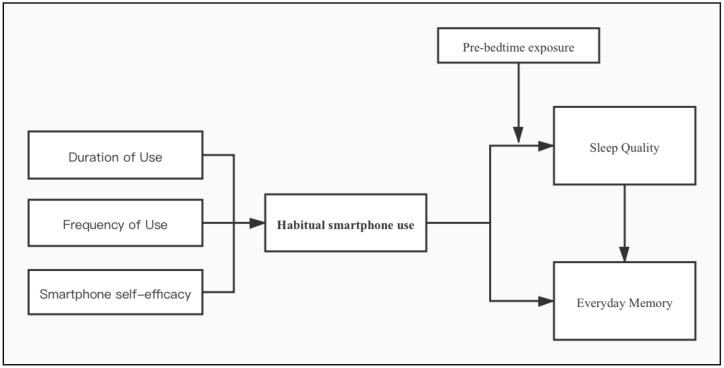
The impact of habitual smartphone use on sleep quality and everyday memory.

To address the limitations of previous research and make new contributions to the knowledge about smartphones’ effects on school-age youths, our study proposes a localized and integrated model based on a nationally representative sample of Chinese students aged 6–18 (*N* = 2298). It aims to: (1) explore the current trends of youths’ smartphone use and any potential gender or school year differences; (2) investigate whether youths’ smartphone behaviors and perceptions (e.g., the duration, frequency of smartphone use, and smartphone self-efficacy) are associated with the habitual smartphone use, and its possible effects on youths’ sleep quality and everyday memory, and (3) find out whether pre-bedtime exposure to smartphones could moderate the association between habitual smartphone use and sleep quality (see Figure 1). The findings should contribute to a better understanding of the impact of smartphone use on the general youth population, especially those who show little prevalence of smartphone addiction.

## 2. Materials and Methods

### 2.1. Sampling

To test the hypotheses, a national mail survey was conducted in China from November 2019 to January 2020 after it was approved by the IRB of the authors’ university. A cluster-randomized sampling with stratification of different areas was used to obtain a nationally representative sample. A total of 2918 students from 63 schools were randomly recruited in 21 provinces and metropolitans across China (i.e., Hunan, Hubei, Henan, Beijing, Hebei, Shanxi, Shanghai, Jiangsu, Shandong, Guangdong, Guangxi, Hainan, Yunnan, Guizhou, Chongqing, Shanxi, Gansu, Xinjiang, Heilongjiang, Jilin, and Liaoning) with a response rate of 78.8% after 620 students’ answers were excluded into the analyses due to missing data. The students came from both urban and rural areas in Central China (14.5%, *n* = 333), North China (15.1%, *n* = 347), East China (14.2%, *n* = 326), South China (12.8%, *n* = 294), Southwest China (16.0%, *n* = 368), Northwest China (14.7%, *n* = 338), and Northeast China (12.6%, *n* = 292). Here “*N*” refers to the entire sample of population, and “*n*” to a sub-group.

### 2.2. Procedure

Before the investigation, we verified the applicability and reliability of the measurement scales used through a thorough literature review. Then we did a pretest among 30 child-parent teams to validate the measures among Chinese youths. Ten students from an elementary school, middle school, or high school, respectively, and one of their parents were invited to participate in the pretest. Focus group interviews were conducted to collect their comments and suggestions after they filled out the questionnaire. Based on their feedback, some problematic items were revised accordingly to improve accuracy and conciseness, and then the scales were finalized for this study. Subsequently, we adopted the cluster-randomized sampling with stratification of different areas to select the target schools. Students in the target schools were surveyed anonymously in their school classrooms. Whenever needed, careful guidance and illustration were offered by their teachers who were fully informed about the survey and research design. Participants completed the survey after informed consent was obtained from the schools, teachers, and participants. Investigational sessions lasted approximately 45 min, in which cross-sectional data were collected.

### 2.3. Measures

#### 2.3.1. Habitual Smartphone Use

Habitual use of smartphone was measured by the 17-item Mobile Phone Addiction Index (MPAI) [24], which includes statements like “*I find it difficult to switch off my mobile phone*”, etc. The scores on the 17 items were averaged to form the index of habitual smartphone use (mean *[M] = 1.87,* standard deviation *[SD] =* 0.80, Cronbach’α = 0.92). Frequency of smartphone use was measured by asking “*How often do you use your smartphone*?” with the answers ranging from 1 *=* not at all, 2 = rarely, 3 = occasionally, 4 = often, 5 = always, to 6 = many times a day (*M* = 3.05, *SD* = 1.42). Duration of use was measured by asking “*How long did you use your smartphone on an average day in the past week*?” with the choices 1 = *never*, 2 = *less than half an hour*, 3 = 0.5–1 h, 4 = 1–2 h, 5 = 2–4 *h,* to 6 = more than 4 h (*M* = 3.58, *SD* = 1.48). Prebedtime exposure was measured with three items, including “I*n the past week, how long did you use your smartphone on average before you go to sleep each day*?” with the answer choices 1 = never, 2 = less than 15 min, 3 = 15–30 min, 4 = 31–60 min, to 5 = more than 60 min, etc.

#### 2.3.2. Smartphone Self-Efficacy

This construct was measured by six items, which were modified based on the scales of power users and ICT power usage [25,26]. The items include “*I make good use of most of the features available in smartphones*”, with the choices 1 *=* not at all true for me, 2 = not very true for me, 3 =neither true nor false for me, 4 = mostly true for me, to 5 = very true for me. The six items were averaged to form the smartphone self-efficacy scale (*M* = 3.25, *SD* = 1.11, Cronbach’α = 0.80).

#### 2.3.3. Sleep Quality

Sleep quality was measured by the 19-item Chinese version of the Pittsburg Sleep Quality Index (PSQI) [27]. We first verified the reliability and validity of the scale on Chinese youth by conducting a thorough literature review of its usage and then a pretest and focus group discussion. Firstly, previous studies indicated that PSQI had been used as a reliable and effective measurement of sleep quality in children, including very young children [28,29,30,31]. Secondly, the feedback from our focus group discussions with the 30 child-parent teams helped improve the scale by removing any confusing items. For example, we removed the items of use of sleep medication, which is inappropriate for young children. The final results showed that all the young participants could understand the meaning of each item and their self-reported answers were consistent in what their parents reported. Thirdly, before and during the investigation, careful guidance and illustration were offered by participants’ teachers who were fully informed and well-trained on this survey. Lastly, the final 19 items measured six indexes of sleep quality: (1) subjective sleep quality; (2) sleep latency; (3) sleep duration; (4) sleep efficiency; (5) sleep disturbance; and (6) sleep dysfunction. Each component weighted equally on a 0–3 scale. The items were added up to form the sleep quality scale (total score ranging between 0 and 18) (*M* = 5.12, *SD* = 2.31). The final data obtained by the 19 items showed good reliability (Cronbach’α = 0.77). A higher score represented worse sleep quality.

#### 2.3.4. Everyday Memory

The 13-item Everyday Memory Questionnaire-revised (EMQ-R) [32] was used to measure everyday memory. Likewise, to ensure the validity of measures, several steps were taken to develop this scale. Firstly, based on previous studies, the revised Everyday Memory Questionnaire consisted of 13 items. Each item described an everyday activity that tested students’ fractionated components of memory, which served as the theoretical foundation of the Everyday Memory Questionnaire-revised used in this study. Secondly, to help children to recall daily memory failures in an easier way, reducing the likelihood of response bias like exaggerating their level of everyday memory, we chose response items with specific frequency claims, such as “*having to check whether you have done something that you should have done*,” with the choices 1 = once or less in the last month, 2 = more than once a month but less than once a week, 3 = about once a week, 4 = more than once a week or less than once a day, to 5 = once or more in a day. Thirdly, pretest showed that even the young participants aged from 6 to12 could understand the statements accurately, and their self-reported answers were also consistent in what their parents reported. Finally, the 13 items were averaged to form the construct of everyday memory (*M* = 2.19, *SD* = 0.83). The 13 items showed good reliability (Cronbach’α = 0.90) with higher scores indicating worse memory.

### 2.4. Statistical Analyses

We first conducted the descriptive statistical analyses with SPSS24.0, and then tested the conceptual model and the moderating effect with AMOS 25.0. The following criteria were used to evaluate the overall fit of the research models: (1) X^2^/df: between 1 and 3; (2) GFI, AGFI: close to 1.00; (3) RESMA: less than 0.05. Starting with the seminal research by Kenny and Judd [33], when all exogenous variables are latent variables and are measured by multiple indicators, structural equation models play an important role in analyzing the interaction effects between variables. We established a latent variable interaction effect model to verify the moderating effect, which has been proven to be an effective method to measure the interaction effects of latent variables [34,35].

## 3. Results

### 3.1. Descriptive Statistics

The sample consisted of 1180 girls and 1108 boys (10 respondents didn’t report their gender), whose ages ranged from 6 to 18 with an average age of 13.3 years (*SD* = 2.385). Among them, 748 students came from elementary schools (37.5%, *M* = 10.75, *SD* = 1.069), 787 from middle schools (32%, *M* = 13.22, *SD* = 1.267), and 763 from high schools (30.5%, *M* = 15.96, *SD* = 0.95). The sample statistics are reported in Table 1.

### 3.2. Aim 1-Gender and School-Year Differences Concerning Smartphone Usage

As shown in Table 1, the analyses showed that Chinese students exhibited habitual smartphone use based on the scale of MPAI, who were generally confident in using their mobile devices, but few had a smartphone addiction (*M* = 1.87, *SD* = 0.80). As Table 2 showed, boys (*M* = 3.11, *SD* = 1.44) spent more time on smartphones than girls (*M* = 2.99, *SD* = 1.40), while girls used the devices significantly more before bedtime (*M* = 2.23, *SD* = 0.89) than boys (*M* = 2.16, *SD* = 0.87). Boys showed a higher level of habitual use (*M* = 1.91, *SD* = 0.81) and smartphone self-efficacy (*M* = 3.38, *SD* = 1.10) than the girls (*M* = 1.84, *SD* = 0.79; *M* = 3.12, *SD* = 1.11). But no significant differences was detected between boys and girls in terms of how frequently they used smartphones (*p* > 0.05).

There are also significant school-year differences in habitual smartphone use between students in elementary, middle, and high schools (*F* = 53.114, *p* < 0.001). High school students demonstrated the higher level of habitual smartphone use (*M* = 2.07, *SD* = 0.86) than both middle school students (*M* = 1.87, *SD* = 0.76) and elementary school students (*M* = 1.66, *SD* = 0.70).

### 3.3. Aim 2- Predictors of Habitual Smartphone Use in Youths

An integrated model (Model 1) was developed to explore the correlates and impacts of youth habitual smartphone use. All the results showed a good model fit (X^2^/df = 2.714, GFI = 0.982, AGFI = 0.973, RMSEA = 0.027). As shown in Table 3, habitual smartphone use could be significantly predicted by duration of use (*β* = 0.323, *SE* = 0.196, *p* < 0.001), frequency of use (*β* = 0.075, *SE* = 0.187, *p* < 0.001), and smartphone self-efficacy (*β* = 0.243, *SE* = 0.044, *p* < 0.001). This implies that Chinese students who used smartphones for a long time with higher frequency demonstrated a stronger habit of smartphone use.

Besides, those reporting higher level of habitual smartphone use were more likely to have a poorer sleep quality (*β* = 0.575, *SE* = 0.013, *p* < 0.001), and a worse everyday memory (*β* = 0.332, *SE* = 0.051, *p* < 0.001). The data also reveal a positive association between sleep quality and memory (*β* = 0.429, *SE* = 0.396, *p* < 0.001).

### 3.4. Aim 3- Moderating Effect of Prebedtime Exposure

**Figure 2:**
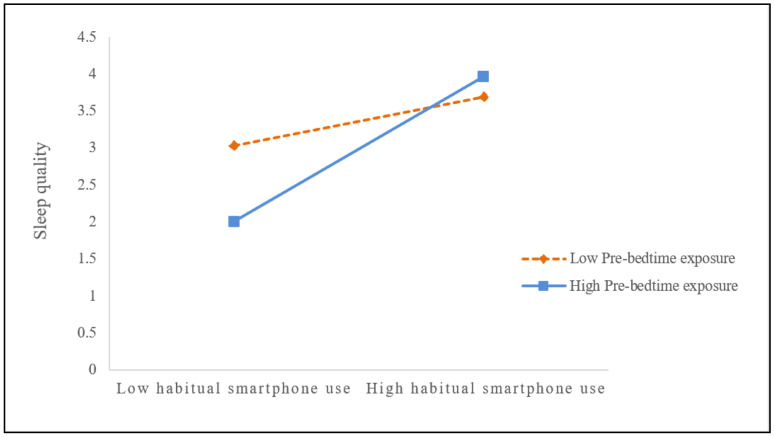
Prebedtime exposure moderated the relationship between habitual smartphone use and sleep quality.

A moderated effect analysis discovers that prebedtime exposure moderated the relationship of habitual smartphone use and sleep quality (Model 2). All the results show a good model fit (X^2^/df = 2.895, GFI = 0.992, AGFI = 0.982, RMSEA = 0.027). As shown in Table 4, both prebedtime exposure and habitual smartphone use had a mediation effect on sleep quality. Results of the bootstrapping procedure reveal that the interaction of habitual smartphone use and prebedtime exposure showed significant effects on sleep quality (*β* = 0.648, *SE* = 0.025, *p* < 0.001). Longer prebedtime exposure significantly strengthened the habitual smartphone use, which consequently hurt sleep latency and sleep efficiency, resulting in poorer sleep quality. The data showed that sleep quality was much worse in those with high prebedtime exposure than those with low exposure (see Figure 2).

## 4. Discussion

### 4.1. Summary of the Results

Given the far-reaching impact of smartphones on school-age children and adolescents, this study investigates the predictors and impacts of habitual smartphone use. Based on a nationally representative sample of Chinese students, it discovers that the youth habitual smartphone use could significantly alter sleep quality and everyday memory. The findings confirm a critical role of smartphones in youths’ lives at home and at school. Boys and high school students were significantly more likely to develop habitual smartphone behavior than girls and those attending primary and middle schools. Besides, the duration, frequency as well as self-efficacy of smartphone use could positively predict the habitual smartphone use, which in turn negatively impacted sleep quality and everyday memory. Moreover, prebedtime exposure of smartphone positively moderated the effect of habitual smartphone use on sleep quality.

### 4.2. Theoretical Implications

This study offers important theoretical implications to the literature on youths’ habitual smartphone use, sleep quality and every memory. First, it advances our knowledge about the habitual smartphone use of Chinese students. Despite many studies focusing on smartphone use of young students within global contexts and developed countries, less is known about the youths’ conditions in developing countries, like China. Some scholars reported that the smartphone use is common among children and adolescents [36,37]. However, measures of smartphone use were not always consistent and the findings varied by investigational scopes [38]. Therefore, it is necessary to conduct a large-scale national survey in China to further validate existing findings. On the basis of a nationally representative sample of Chinese students, this study has discovered that the duration and the frequency of smartphone use are positively associated with the habitual smartphone use.

As media dependency theory illustrates the cognitive, behavioral and affective consequences of media use [39], previous studies paid much attention to the impacts of smartphone use on behaviors, such as improper phone use [40], purchase behavior [41], academic performance [42] and so on. Going beyond the existing literature, our study centers on the habitual use of smartphone among the youth in a developing country. It examines the extent of habitual smartphone use among school children from four dimensions, i.e., inability to control craving, feeling anxious or lost, withdrawal, and productivity loss. In agreement with the findings from other countries [43], this study uncovers the habitual smartphone use among Chinese youths (*M* = 1.87), but most of them did not show the tendency of smartphone addiction. Two possible reasons may help interpret this finding. Firstly, this study used a nationally representative sample including school children from elementary, middle, and high schools, who were younger than the samples in other studies that recruited college students only [1]. Unlike in colleges, most Chinese elementary and middle schools impose strict limitations on smartphone use by students, which could make the students less likely to develop addictive phone behaviors. Secondly, parenting mediation played an essential role in children’s media use [44]. Most Chinese parents tended to limit their children’s smartphone use due to the heavy school workload and fierce academic competition.

Secondly, our findings add to the growing literature that school-age children with higher level of smartphone self-efficacy reported more positive effects of smartphone usage, which should be taken into account in future digital literacy education. It’s clear that self-control is needed to cope with complex smartphone technologies. Thus, on the basis of existing research of ICT self-efficacy [25,45], this study proposes a new variable of “smartphone self-efficacy” and identifies its association with smartphone dependence for the first time. School children with a higher smartphone self-efficacy were found to make better use of smartphones in general.

Third, this work broadens the scholarly knowledge on the consequences of youths’ habitual smartphone use. Previous studies have separately examined the relationship between smartphone use and sleep quality [17,46,47] or memory [20,21]. Our study establishes an integrated model of youth habitual smartphone use, sleep quality, and memory performance, which extends previous findings mainly based on the smartphone use among adults or college students [46,48,49]. Given that too much time spent in media activities could affect children’s cognitive, emotional, and health-related development [50], this study adds empirical evidence to the negative impact of habitual smartphone use on children’s sleep quality and everyday memory. It serves as a timely reminder for parents and educators to protect school children from adverse effects of smartphone use.

Fourth, it provides novel information regarding smartphone use in specific contexts. Prebedtime exposure deserves more research effort as it was found to moderate the relationship between students’ habitual smartphone use and sleep quality. Facing heavy school workloads and explicit prohibition of smartphone use, students had little chance to use it as much as they wished during the day [51]. As a result, they might spend some time on smartphone at night, especially before bedtime after all homework was done. This habit could potentially cause chronic sleep deprivation. The students who used smartphone more often before bedtime usually slept less with poorer sleep quality and more daytime fatigue. Existing studies showed that there were two possible mechanisms through which smartphone dependence could cause sleep disruption. One was melatonin suppression due to exposure to bright light from screens [52,53], the other was sleep disturbance due to the emotional arousal brought by various information received prebedtime [37,54]. Overall, if prebedtime exposure of smartphone became routine, sleep quality would significantly be affected.

### 4.3. Practical Implications

The findings suggest that a moderate amount of smartphone use, instead of habitual use, might be crucial in preventing negative health outcomes. Prior studies showed that the most common reason for children to get a smartphone from parents is to stay connected with family members [55]. As a multifunctional tool, smartphones are used like a minicomputer by students for both educational and entertainment purposes, in addition to staying in touch with parents and friends. Obviously, today’s youth are not only “connected” with others and information, but also feel insecure or fear they will miss something important when not connected [56]. This may explain part of the habitual use of smartphones among Chinese students. As smartphone-based activities can be time-consuming, parents and educators need to become fully aware of the imperceptible impacts of smartphones and take their responsibility in regulating improper smartphone use of youths. Besides, youths’ prebedtime exposure of smartphone need to be strictly administrated and controlled by their parents and themselves, considering the significant moderating effect revealed in this study.

### 4.4. Limitations and Future Studies

Interpretation of the results in the present research requires attention to several limitations it has. First, despite a national sample of students being used, the results have limited generalizability, some of which may not be applied to the youth living in other countries. Second, some cultural and social features in China need to be factored in the data inference. Third, the cross-sectional study design makes it difficult to test the causal relationships between smartphone habitual usage and sleep disturbance, everyday memory. The research design also makes it hard to determine whether the outcome of poor sleep quality followed exposure to smartphone content or the exposure resulted from the outcome. Fourth, this study did not specifically analyze the effect of sleep disorder, which could play an important role in the dynamic association between smartphone use and sleep quality in youths. Finally, this study does not consider the specific ways of smartphone usage in the youth. Some differences may exist when they use the device for different purposes, such as an education tool, or messaging, gaming, tweeting, video watching, sexting, etc.

The MPAI values out of this study should be compared with other studies involving smartphone addiction, which may generate more insights concerning youth use of smartphone. Although smartphone has made certain school-related tasks easier, some types of smartphone usage are discouraged or even banned by teachers and parents, which could place a series of stressors on some of the youth. Future studies can make progress in the above aspects.

As the extent of school children’s habitual smartphone use varies from country to country, future studies may examine the cross-cultural differences in the youth smartphone use in multiple countries. They may also compare the levels and impacts of habitual smartphone use among different populations (e.g., children vs. adolescents; adolescents vs. adults), which can help to explore the influence of demographics on the extent and effects of smartphone dependence. As smartphone use becomes an important component of children’s social ecology [57], it will be imperative to examine the long-term effects of smartphone experience on school children’s cognitive development and health.

## 5. Conclusions

Mobile phone usage is reshaping younger generations’ daily lives and becoming a habit for more and more children and adolescents. This study reveals that Chinese youth’s habitual smartphone use has a significant impact on their sleep quality and everyday memory, even when they did not develop smartphone addiction. The findings suggest that youth habitual smartphone use may be one of the critical factors that affect their health, cognition and beyond, though many of them exhibited no addictive use. Habits developing during childhood and adolescence could have a considerable impact on their adult lives. The results should contribute to a better understanding of the smartphone impact on school-age youths, which lays the groundwork of mitigating detrimental effect of smartphone usage in children and adolescents. This work thus provides theoretical and practical references in media technology education and children’s media use evaluation.

## Figures and Tables

**Table 1:** Descriptive statistics.

Variables	Range	*M*	*SD*	*Median*	*Mode*
Duration of usage	1–6	3.05	1.42	3.00	2.00
Frequency of usage	1–6	3.57	1.47	3.00	3.00
Smartphone self-efficacy	1–5	3.25	1.11	3.40	3.00
Habitual smartphone use	1–5	1.87	0.80	1.65	1.00
Sleep quality	0–18	5.12	2.31	5.00	4.00
Everyday memory	1–5	2.19	0.83	2.08	1.77
Prebedtime exposure	1–5	2.20	0.88	2.33	2.33

Note. The range of some variables only represented the degree of change, not the actual numerical meaning, including duration of usage, frequency of usage, and prebedtime exposure.

**Table 2:** Gender and school-year differences in smartphone use.

Variables	Female *M(SD)* (*n* = 1180)	Male *M(SD)* (*n* = 1108)	*t*	Elementary School *M(SD)* (*n* = 748)	Middle School *M(SD)* (*n* = 787)	High School *M (SD)* (*n* = 763)	*F*
Duration of usage	2.99 (1.40)	3.11 (1.44)	−2.061 *	2.95 (1.34)	3.19 (1.38)	3.01 (1.52)	5.588 ***
Frequency of usage	3.53 (1.45)	3.61 (1.51)	−1.243	3.56 (1.37)	3.74 (1.42)	3.42 (1.63)	9.219 ***
Smartphone self-efficacy	3.12 (1.11)	3.38 (1.10)	−5.707 ***	2.83 (1.16)	3.34 (1.03)	3.56 (1.01)	83.288 ***
Habitual smartphone use	1.84 (0.79)	1.91 (0.81)	−2.158 *	1.66 (0.70)	1.87 (0.76)	2.07 (0.86)	53.114 ***
Sleep quality	5.16 (2.33)	5.07 (2.29)	1.008	4.14 (2.18)	5.16 (2.26)	6.02 (2.11)	140.624 ***
Everyday memory	2.21 (0.84)	2.17 (0.82)	1.196	1.96 (0.79)	2.31 (0.87)	2.30 (0.80)	47.756 ***
Prebedtime exposure	2.23 (0.89)	2.16 (0.87)	2.061 *	1.93 (0.78)	2.20 (0.86)	2.45 (0.90)	68.264 ***

Note: * *p* < 0.05. *** *p* < 0.001. Ten respondents didn’t report their gender.

**Table 3:** An integrated model to reveal the correlates of habitual smartphone use and its impact on sleep quality and everyday memory (Model 1).

Model 1	*β*	*S.E.*	*t*
DV: Habitual smartphone use			
IV: Duration of use	0.323 ***	0.196	15.721
IV: Frequency of use	0.075 ***	0.187	3.657
IV: Self-efficacy	0.243 ***	0.044	9.587
IV: Habitual smartphone use			
DV: Sleep quality	0.575 ***	0.013	12.636
DV: Everyday memory	0.332 ***	0.051	10.142
IV: Sleep quality			
DV: Everyday memory	0.429 ***	0.396	14.076

Note. Bootstrap sample size = 5000. *** *p* < 0.001. Ten respondents didn’t report their gender. For sleep quality and memory, the higher score represents worse sleep quality and memory.

**Table 4:** Prebedtime exposure of smartphone as a moderator of habitual smartphone use and sleep quality (Model 2).

Model 2	*β*	*S.E.*	*t*
DV: Sleep quality			
IV: Habitual smartphone use	0.329 ***	0.008	6.792
IV: Prebedtime exposure	−0.373 ***	0.062	−4.955
IV: Habitual smartphone use*Prebedtime exposure	0.648 ***	0.025	3.701
**Standardized Regression Weights**	***β***	**Lower**	**Upper**
M –1 SD	−0.463 *	−1.098	−0.049
M	0.338 ***	0.232	0.445
M +1 SD	1.138 ***	0.676	1.802

Note: Bootstrap sample size = 5000. * *p* < 0.05. *** *p* < 0.001. Ten respondents didn’t report their gender. For sleep quality and memory, the higher score represents worse sleep quality and memory.

## Data Availability

The data presented in this study are available on request from the corresponding author. The data are not publicly available due to the physical privacy of the participants.

## References

[1] Li L., Gao H.Y., Xu Y.H. (2020). The mediating and buffering effect of academic self-efficacy on the relationship between smartphone addiction and academic procrastination. Comput. Educ..

[2] Grontved A., Ried-Larsen M., Moller N.C., Kristensen P.L., Wedderkopp N., Froberg K., Hu F.B., Ekelund U., Andersen L.B. (2014). Youth screen-time behaviour is associated with cardiovascular risk in young adulthood: The European Youth Heart Study. Eur. J. Prev. Cardiol..

[3] Anderson M., Jiang J. (2018). Teens, Social Media & Technology 2018.

[4] Annual Report on the Internet Use and Reading Practice of Chinese Minors (2017–2018). https://www.ssap.com.cn/c/2018-09-10/1071885.shtml.

[5] Anshari M., Alas Y., Hardaker G., Jaidin J.H., Smith M., Ahad A.D. (2016). Smartphone habit and behavior in Brunei: Personalization, gender, and generation gap. Comput. Hum. Behav..

[6] Pan Y.-C., Chiu Y.-C., Lin Y.-H. (2020). Systematic review and meta-analysis of epidemiology of internet addiction. Neurosci. Biobehav. Rev..

[7] Sohn S., Rees P., Wildridge B., Kalk N.J., Carter B. (2019). Prevalence of problematic smartphone usage and associated mental health outcomes amongst children and young people: A systematic review, meta-analysis and GRADE of the evidence. BMC Psychiatry.

[8] Wohn D.Y., Ahmadi M. (2019). Motivations and habits of micro-news consumption on mobile social media. Telemat. Inf..

[9] Wood W., Runger D. (2016). Psychology of Habit. Annu. Rev. Psychol..

[10] van Deursen A.J.A.M., Bolle C.L., Hegner S.M., Kommers P.A.M. (2015). Modeling habitual and addictive smartphone behavior The role of smartphone usage types, emotional intelligence, social stress, self-regulation, age, and gender. Comput. Hum. Behav..

[11] Andone I., Blaszkiewicz K., Eibes M., Trendafilov B., Montag C., Markowetz A. How age and gender affect smartphone usage. Proceedings of the 2016 ACM international joint conference on pervasive and ubiquitous computing.

[12] Yang S.Y., Lin C.Y., Huang Y.C., Chang J.H. (2018). Gender differences in the association of smartphone use with the vitality and mental health of adolescent students. J. Am. Coll. Health.

[13] Bandura A. (1991). Social cognitive theory of self-regulation. Organ. Behav. Hum. Decis. Process..

[14] Bandura A. (2004). Health promotion by social cognitive means. Health Educ. Behav..

[15] Zhang M.X., Wu A.M.S. (2020). Effects of smartphone addiction on sleep quality among Chinese university students: The mediating role of self-regulation and bedtime procrastination. Addict. Behav..

[16] Ward A.F., Duke K., Gneezy A., Bos M.W. (2017). Brain Drain: The Mere Presence of One’s Own Smartphone Reduces Available Cognitive Capacity. J. Assoc. Consum. Res..

[17] Van den Bulck J. (2003). Text messaging as a cause of sleep interruption in adolescents, evidence from a cross-sectional study. J. Sleep Res..

[18] Kamibeppu K., Sugiura H. (2005). Impact of the mobile phone on junior high-school students’ friendships in the Tokyo metropolitan area. Cyberpsychol. Behav..

[19] Smith C., de Wilde T., Taylor R.W., Galland B.C. (2020). Prebedtime screen sse in adolescents: A survey of habits, barriers, and perceived acceptability of potential interventions. J. Adolesc. Health..

[20] Fjortoft N., Gettig J., Verdone M. (2018). Smartphones, Memory, and Pharmacy Education. Am. J. Pharm. Educ..

[21] Liebherr M., Schubert P., Antons S., Montag C., Brand M. (2020). Smartphones and attention, curse or blessing?—A review on the effects of smartphone usage on attention, inhibition, and working memory. Comput. Hum. Behav. Rep..

[22] Walker M.P., Stickgold R. (2010). Overnight alchemy: Sleep-dependent memory evolution. Nat. Rev. Neurosci..

[23] Rasch B., Born J. (2013). About Sleep’s Role in Memory. Physiol. Rev..

[24] Leung L., Konijn E.A., Tanis M.A., Utz S., Konijn E.A., Utz S., Tanis M., Barnes S.B. (2008). Leisure boredom, sensation seeking, self-esteem, addiction symptoms and patterns of mobile phone use. Mediated interpersonal communication.

[25] Zhong B., Appelman A.J. (2014). How college students read and write on the web: The role of ICT use in processing online information. Comput. Hum. Behav..

[26] Zhong B., Yang F. (2018). How we watch TV tomorrow: Viewers’ perception towards interactivity functions on smart TV. Int. J. Asian Bus. Inf. Manag..

[27] Buysse D.J., Reynolds C.F., Monk T.H., Berman S.R., Kupfer D.J. (1989). The Pittsburgh Sleep Quality Index: A new instrument for psychiatric practice and research. Psychiatry Res..

[28] Brand S., Blechschmidt A., Muller A., Sader R., Schwenzer-Zimmerer K., Zeilhofer H.F., Holsboer-Trachsler E. (2009). Psychosocial functioning and sleep patterns in children and adolescents with cleft lip and palate (CLP) compared with healthy controls. Cleft. Palate Cran J..

[29] Llabre M.M., Hadi F. (2009). War-related exposure and psychological distress as predictors of health and sleep: A longitudinal study of Kuwaiti children. Psychosom. Med..

[30] Liu Q.Q., Zhou Z.K., Yang X.J., Kong F.C., Niu G.F., Fan C.Y. (2017). Mobile phone addiction and sleep quality among Chinese adolescents: A moderated mediation model. Comput. Hum. Behav..

[31] Tan L.P. (2004). The effects of background music on quality of sleep in elementary school children. J. Music.

[32] Royle J., Lincoln N.B. (2008). The everyday memory questionnaire-revised: Development of a 13-item scale. Disabil. Rehabil..

[33] Kenny D.A., Judd C.M. (1984). Estimating the nonlinear and interactive effects of latent variables. Psychol. Bull..

[34] Marsh H.W., Wen Z.L., Hau K.T. (2004). Structural equation models of latent interactions: Evaluation of alternative estimation strategies and indicator construction. Psychol. Methods..

[35] Saris W.E., Batista-Foguet J.M., Coenders G. (2007). Selection of indicators for the interaction term in structural equation models with interaction. Qual. Quant..

[36] Munezawa T., Kaneita Y., Osaki Y., Kanda H., Minowa M., Suzuki K., Higuchi S., Mori J., Yamamoto R., Ohida T. (2011). The association between use of mobile phones after lights out and sleep disturbances among Japanese adolescents: A nationwide cross-sectional survey. Sleep.

[37] Vernon L., Modecki K.L., Barber B.L. (2018). Mobile Phones in the Bedroom: Trajectories of sleep habits and subsequent adolescent psychosocial development. Child Dev..

[38] Vrijheid M., Armstrong B.K., Bedard D., Brown J., Deltour I., Iavarone I., Krewski D., Lagorio S., Moore S., Richardson L. (2009). Recall bias in the assessment of exposure to mobile phones. J. Expo Sci. Env. Epid..

[39] Ball-Rokeach S.J., DeFleur M.L. (1976). A dependency model of mass-media effects. Commun. Res..

[40] Lin TT C., Chiang Y.H. (2017). Investigating predictors of smartphone dependency symptoms and effects on academic performance, improper phone use and perceived sociability. Int. J. Mob. Commun..

[41] Ting D.H., Lim S.F., Patanmacia T.S., Low C.G., Ker G.C. (2011). Dependency on smartphone and the impact on purchase behaviour. Young Consum..

[42] Samaha M., Hawi N.S. (2017). Relationships among smartphone addiction, stress, academic performance, and satisfaction with life (vol 57, pg 321, 2016). Comput. Hum. Behav..

[43] Stankovic M., Nesic M., Cicevic S., Shi Z.H. (2021). Association of smartphone use with depression, anxiety, stress, sleep quality, and internet addiction. Empirical evidence from a smartphone application. Pers. Indiv. Differ..

[44] Padilla-Walker L.M., Coyne S.M., Fraser A.M., Dyer W.J., Yorgason J.B. (2012). Parents and adolescents growing up in the digital age: Latent growth curve analysis of proactive media monitoring. J. Adolesc..

[45] Zhong B. (2013). From smartphones to iPad: Power users’ disposition toward mobile media devices. Comput. Hum. Behav..

[46] Demirci K., Akgonul M., Akpinar A. (2015). Relationship of smartphone use severity with sleep quality, depression, and anxiety in university students. J Behav Addict.

[47] Spagnoli P., Balducci C., Fabbri M., Molinaro D., Barbato G. (2019). Workaholism, Intensive Smartphone Use, and the Sleep-Wake Cycle: A Multiple Mediation Analysis. Int. J. Env. Res. Pub. Health.

[48] Bianchi A., Phillips J.G. (2005). Psychological predictors of problem mobile phone use. Cyberpsychol. Behav..

[49] Sahin S., Ozdemir K., Unsal A., Temiz N. (2013). Evaluation of mobile phone addiction level and sleep quality in university students. Pak. J. Med. Sci..

[50] Wright J.C., Huston A.C., Murphy K.C., St Peters M., Pinon M., Scantlin R., Kotler J. (2001). The relations of early television viewing to school readiness and vocabulary of children from low-income families: The early window project. Child Dev.

[51] Gao Q., Yan Z., Zhao C., Pan Y., Mo L. (2014). To ban or not to ban: Differences in mobile phone policies at elementary, middle, and high schools. Comput. Hum. Behav..

[52] Cajochen C., Frey S., Anders D., Spati J., Bues M., Pross A., Mager R., Wirz-Justice A., Stefani O. (2011). Evening exposure to a light-emitting diodes (LED)-backlit computer screen affects circadian physiology and cognitive performance. J. Appl. Physiol..

[53] Wood B., Rea M.S., Plitnick B., Figueiro M.G. (2013). Light level and duration of exposure determine the impact of self-luminous tablets on melatonin suppression. Appl. Erg..

[54] Oshima N., Nishida A., Shimodera S., Tochigi M., Ando S., Yamasaki S., Okazaki Y., Sasaki T. (2012). The Suicidal Feelings, Self-Injury, and Mobile Phone Use After Lights Out in Adolescents. J. Pediatr. Psychol..

[55] Derevensky J.L., Hayman V., Gilbeau L. (2019). Behavioral addictions excessive gambling, gaming, internet, and smartphone use among children and adolescents. Pediatr. Clin. N. Am..

[56] Gentina E., Rowe F. (2020). Effects of materialism on problematic smartphone dependence among adolescents: The role of gender and gratifications. Int. J. Inf. Manag..

[57] Calvert S.L., Wilson B.J. (2008). Handbook of Children, Media, and Development.

